# Progress and Challenges Toward Effective Flexible Perovskite Solar Cells

**DOI:** 10.1007/s40820-023-01165-8

**Published:** 2023-08-31

**Authors:** Xiongjie Li, Haixuan Yu, Zhirong Liu, Junyi Huang, Xiaoting Ma, Yuping Liu, Qiang Sun, Letian Dai, Shahzada Ahmad, Yan Shen, Mingkui Wang

**Affiliations:** 1 Wuhan National Laboratory for Optoelectronics, Huazhong University of Science and Technology, 1037 Luoyu Road, Wuhan, 430074 Hubei People’s Republic of China; 2 BCMaterials, Basque Center for Materials, Applications and Nanostructures, University of Basque Country Science Park, 48940 Leioa, Spain; 3 Ikerbasque, Basque Foundation for Science, 48009 Bilbao, Spain

**Keywords:** Flexible photovoltaics, Perovskite, Internal stress, Flexural endurance, Self-healing, Long-term stability

## Abstract

The demand for building-integrated photovoltaics and portable energy systems based on flexible photovoltaic technology such as perovskite embedded with exceptional flexibility and a superior power-to-mass ratio is enormous. The photoactive layer, i.e., the perovskite thin film, as a critical component of flexible perovskite solar cells (F-PSCs), still faces long-term stability issues when deformation occurs due to encountering temperature changes that also affect intrinsic rigidity. This literature investigation summarizes the main factors responsible for the rapid destruction of F-PSCs. We focus on long-term mechanical stability of F-PSCs together with the recent research protocols for improving this performance. Furthermore, we specify the progress in F-PSCs concerning precise design strategies of the functional layer to enhance the flexural endurance of perovskite films, such as internal stress engineering, grain boundary modification, self-healing strategy, and crystallization regulation. The existing challenges of oxygen-moisture stability and advanced encapsulation technologies of F-PSCs are also discussed. As concluding remarks, we propose our viewpoints on the large-scale commercial application of F-PSCs.

## Highlights

- Critical issues including mechanical stability, water and oxygen resistance, transparent electrodes for flexible perovskite solar cells are discussed.
- Roll-to-Roll technology presents a promising avenue for fabrication of flexible perovskite solar cells fabricated for large-scale commercial application.
- Balancing the transmittance and conductivity of transparent electrodes has become a significant issue in developing efficient flexible perovskite solar cells.

## Graphical abstract

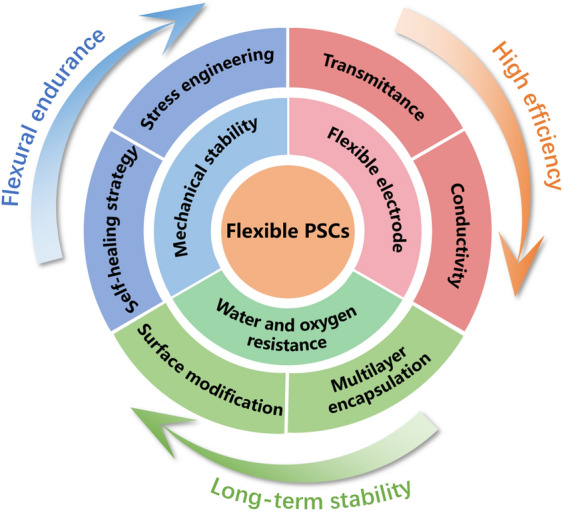

## Introduction

Currently, different types of solar photovoltaic systems have been proposed to effectively convert photons into electricity, for example perovskite solar cells (PSCs) [1–3], organic photovoltaics (OPVs) [4–6], CIGS solar cells [7–9], silicon solar cells [10, 11], CdTe solar cells [12, 13], dye-sensitized solar cells (DSCs) [14, 15], GaAs solar cells [16, 17], and quantum dot solar cells (QDSCs) [18, 19]. For photovoltaic devices, the figure of merit for large-scale application is their efficiency, manufacturing cost, and stability. PSC is endorsed as an emerging technology for photovoltaic applications because of its superior efficiency, facile processable features, and low cost [20].

**Fig. 1:**
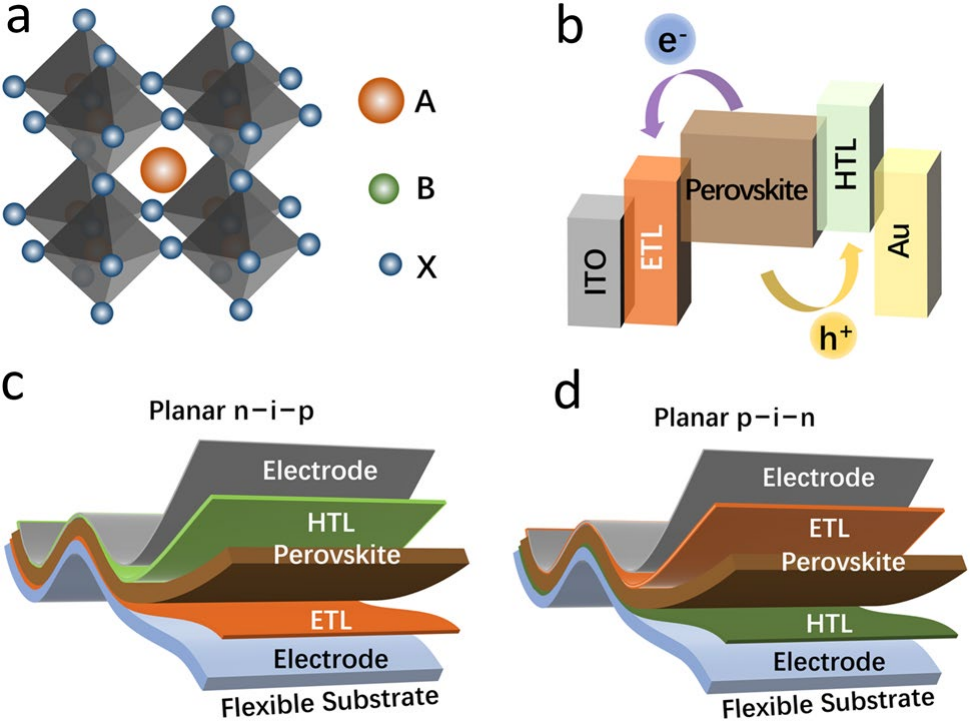
**a** Illustration of a typical crystalline structure of perovskite ABX_3_. **b** Respective energy level and charge transport diagram. Typical flexible PSC devices with **c** n-i-p architecture and **d** p-i-n architecture

These typical hybrid perovskite materials have an octahedral crystalline structure and the general formula ABX_3_ (Fig. 1a). Recently, PCE over 25.8% has been realized in single-junction PSCs [21, 22], stemming from distinctive performances of halide perovskites, such as a tunable bandgap, easy fabrication with low-temperature solution methods, and an excellent absorption coefficient [23]. Further, continuous large-area roll-to-roll manufacturing methods have enabled massive production to substantially enhance the cost-effectiveness of flexible PSCs and exhibit high market prospects [24–26].

Figure 1b presents the energy levels and charge transportation in flexible perovskite solar cells (F-PSC). The photogenerated electron–hole pairs in perovskite layer separate easily to free carriers because of a reduced exciton binding energy. These free charge carriers can be efficiently extracted from the absorber to the electron transport layer (ETL) and hole transport layer (HTL), respectively. According to the direction of light incidence, there are two types of F-PSCs such as regular n-i-p and inverted p-i-n architectures (Fig. 1c, d). The ETL, acting as the charge-selective layer, is deposited on the transparent conductive plastic substrates, followed by fabricating a perovskite absorbing layer with spin-coating technique. Subsequently, the HTL and the back electrodes are deposited to build a regular n-i-p structure planar F-PSCs. In addition, for an inverted p-i-n planar F-PSCs, the positions of the charge transport layers are interchanged [27, 28].

F-PSCs can be made with plastic substrates like PET [29], PEN [30], and PI [31] owing to their naturally flexible nature and low-temperature processing. This fits them in different applications, such as flexible portable power supplies, flexible display devices, and building-integrated photovoltaic (BIPV) systems [32–37]. Notably, F-PSCs can be manufactured via roll-to-roll technology as new paper printing and have been demonstrated for commercial organic PVs [38]. Thus, F-PSCs are widely applied in the domains of wearable devices and portable power sources. They can function as a power source for smartphones, even in indoor ambient light environments, while enabling outdoor operation of portable devices. Additionally, lightweight F-PSCs present highly advantageous conditions for military applications, particularly in individual combat and aerospace settings, where weight reduction is paramount due to constrained payload capacity. In such circumstances, they can reach locations inaccessible to rigid devices. For example, F-PSCs with high power-per-weight ratios demonstrate remarkable potential as power sources for military detection and micro-spacecraft, as well as for environmental and industrial monitoring, utilizing solar leaves or weather balloons.

**Fig. 2:**
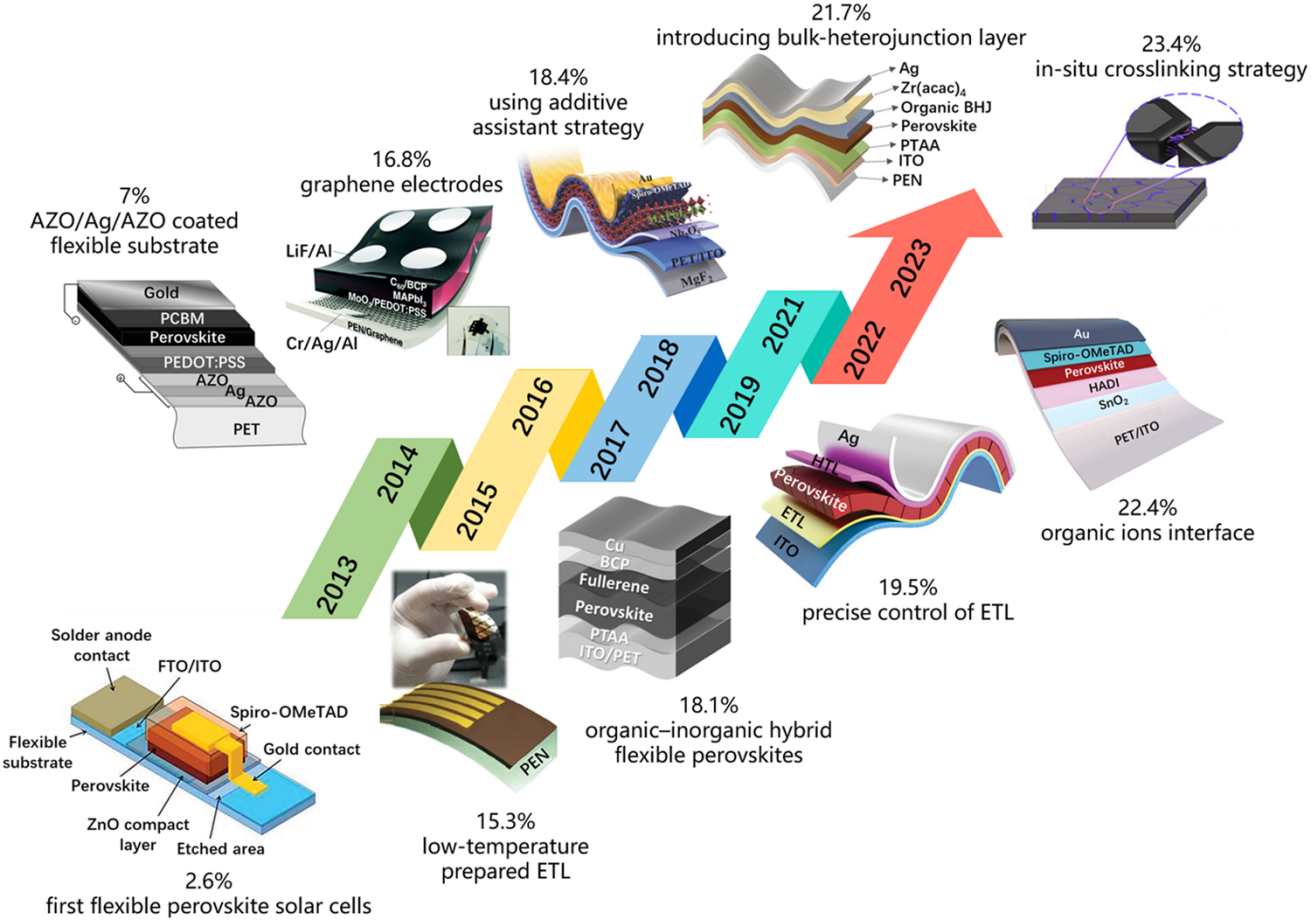
The journey of efficiency evolution for F-PSCs during the period from 2013 to 2023 [39–48]

The first report of the F-PSCs deals with a PCE of 2.62% [39], in which the chemical bath deposition (CBD)-processed ZnO nanorods were used as electron transport layers (Fig. 2). Since then, various routes have been utilized to enhance PCE and flexural endurance of flexible photovoltaics including optimization of the interface and architecture of devices. Among these efforts, the interface modification and optimization of charge transport layers significantly improve the power conversion efficiency and long-term stability for F-PSCs. PET-based conductive substrates made of an aluminum-doped ZnO were also reported for F-PSC [40]. Specifically, the flexible device could endure 50 bending cycles with 99% of the initial PCE. In order to highlight the benefits of F-PSCs, low-temperature processing has been investigated by utilizing conductive metal oxides as charge transportation layers. A low-temperature deposition method was proposed to fabricate highly dispersed Zn_2_SnO_4_ (ZSO) nanoparticles as an electron-conducting electrode for flexible devices with a PCE of 15.3% [41].

In this respect, as a critical functional layer of F-PSCs, the components of perovskite materials are critical in the photovoltaics properties and mechanical reliability. A novel sulfur-based organic molecule to optimize the perovskites system on flexible devices [44] obtained a high efficiency of 18.4%. Nevertheless, the conversion efficiency of Pb-based perovskite devices is limited by their low spectral response in the near-infrared (NIR) wavelength region. Zhu et al. [46] introduced a hetero-junction into flexible devices. The resultant F-PSCs obtained an excellent efficiency of 21.73% and retained 95% of their initial PCE values after 1,000 cycles of consecutive bending. Recently, a novel histamine diiodate (HADI) additive was designed to passivate the buried perovskite surface, achieving a record PCE of 22.4% with over 90% initial PCE maintenance after varied bending cycles [47]. Followed by another report, where flexible photovoltaics achieved a champion efficiency of 23.4% by using in situ cross-linking organic molecules along with perovskite crystal growth [48]. The resultant flexible perovskite films have low Young’s modulus and high crystalline quality, resulting in a maintenance of approximately 90% of their initial PCE for the robust F-PSCs.

In recent years, there has been relevant literature summarizing recent developments in flexible photovoltaic devices [49–52]. Unlike most existing reviews, this report focuses on the key scientific challenges underlying the long-term stability issue in F-PSCs, such as mechanical durability and water-oxygen resistance. Recently, critical progress has been reported to enhance the photovoltaic performances of F-PSCs, achieving the certified PCE of over 23% for single-junction devices [28, 48], which is comparable to those of rigid photovoltaic. To date, extensive study has focused on the strategies to enhance the PCE of F-PSCs. Mechanical stability and flexural endurance are equally important to power conversion efficiency to achieve large-scale applications of F-PSCs (Table 1). Mechanical durability represents the occurrence of cracks on the perovskite layer because of external mechanical stress, which would induce elastic deformation and lattice distortion when twisting, bending, and internal residual stress during thermal annealing. The cracks and lattice distortion on the perovskite layer could generate unfavorable phase transitions and accelerate serious ion migrations in F-PSCs that can eventually result in perovskite decomposition and damage the device performance largely.

**Table 1:** Performance parameters of reported F-PSCs

Device structure	Initial PCE (%)	Bending cycles	Bending radius (mm)	% of initial PCE	Refs.
PEN/ITO/SnO_2_/perovskite/Spiro-OMeTAD/Ag	19.51	6,000	8	95	[45]
PEN/ITO/PTAA/perovskite/CH1007/PCBM/BCP/Ag	21.73	1,000	5	95	[46]
PEN/ITO/HADI-SnO_2_/FA_0.9_Cs_0.1_PbI_3_/Spiro-OMeTAD/Au	22.44	1,000	5	90	[47]
PET/ITO/SnO_2_/perovskite/Spiro-OMeTAD/Au	23.40	5,000	5	93	[48]
PET/ITO/PEDOT:EVA/Perovskite/PCBM/BCP/Ag	19.87	7,000	5	95	[53]
PET/ITO/FI-SnO_2_/Perovskite/Spiro-OMeTAD/Au	21.00	20,000	5	80	[54]
PEN/ITO/PTAA/Perovskite/C60/BCP/Cu	21.76	25,000	5	90	[55]
PEN/ITO/PTAA/PFN-Br/Perovskite/C60/BCP/Cu	20.00	10,000	2.5	73	[56]
PEN/ITO/NiO_x_/Perovskite/PCBM/BCP/Bi_2_Te_3_	18.16	1,000	4	95	[57]
Mica/ITO/PEDOT:PSS/Perovskite/PCBM/BCP/Ag	18.00	5,000	5	92	[58]
PEN/ITO/SnO_2_/Perovskite/Spiro-OMeTAD/Ag	23.10	2,000	10	90	[59]
PET/ITO/SnO_2_/Perovskite/Spiro-OMeTAD/Au	17.98	1,000	2	82	[60]
PET/ITO/NiOx/Perovskite/PCBM/BCP/Ag	19.03	3,000	5	80	[61]

Large-area F-PSCs could be significant for accelerating the commercialization of F-PSCs. Significant achievement has been obtained in producing large-scale F-PSCs currently; however, issues are hindering to achieve commercialization and applications of F-PSCs. For example, it is difficult to manufacture low roughness and homogeneous perovskite films onto flexible substrates featuring rough and inhomogeneous surfaces. Particularly, during the heat sintering procedure of device fabrication, the difference in thermal conductivity between the plastic substrates and the conventional rigid glass substrates might explain the nonuniform perovskite films on the former. Similarly, the conductivity and transportation of electrodes are also essential for efficient and stable photovoltaic devices necessary for commercialization. Arguably, a high level of flexibility with competitive mechanical stability is required to meet the current market demands.

So far, ITO and AZO are the preferred metallic oxides for flexible conductive electrodes. Still, the brittleness of those materials hinders the flexural endurance of flexible photovoltaics. Most of the substrates made of plastics are unable to withstand high-temperature treatments due to their low heat distortion temperature. High annealing temperatures can cause the deformation of flexible substrates, leading to defects in the perovskite layer and adjacent functional layers. This poses a significant challenge in attaining outstanding performances in F-PSCs. Furthermore, the flexible substrates that consist of polymers have weak water and oxygen resistance compared to rigid glass because of a high water vapor transmission rate (WVTR). Thus, the flexible substrates and functional layers should be finely designed to realize efficient and stable large-area F-PSCs.

We review the recent progress on the strategies for improving the devices’ flexural endurance based on perovskite engineering and interfacial modification to induce long-term operational stability. Efforts in the advanced encapsulation strategies are also discussed to solve the problem of penetration of moisture and oxygen through polymer-based flexible substrates.

## Required Properties of F-PSCs

**Fig. 3:**
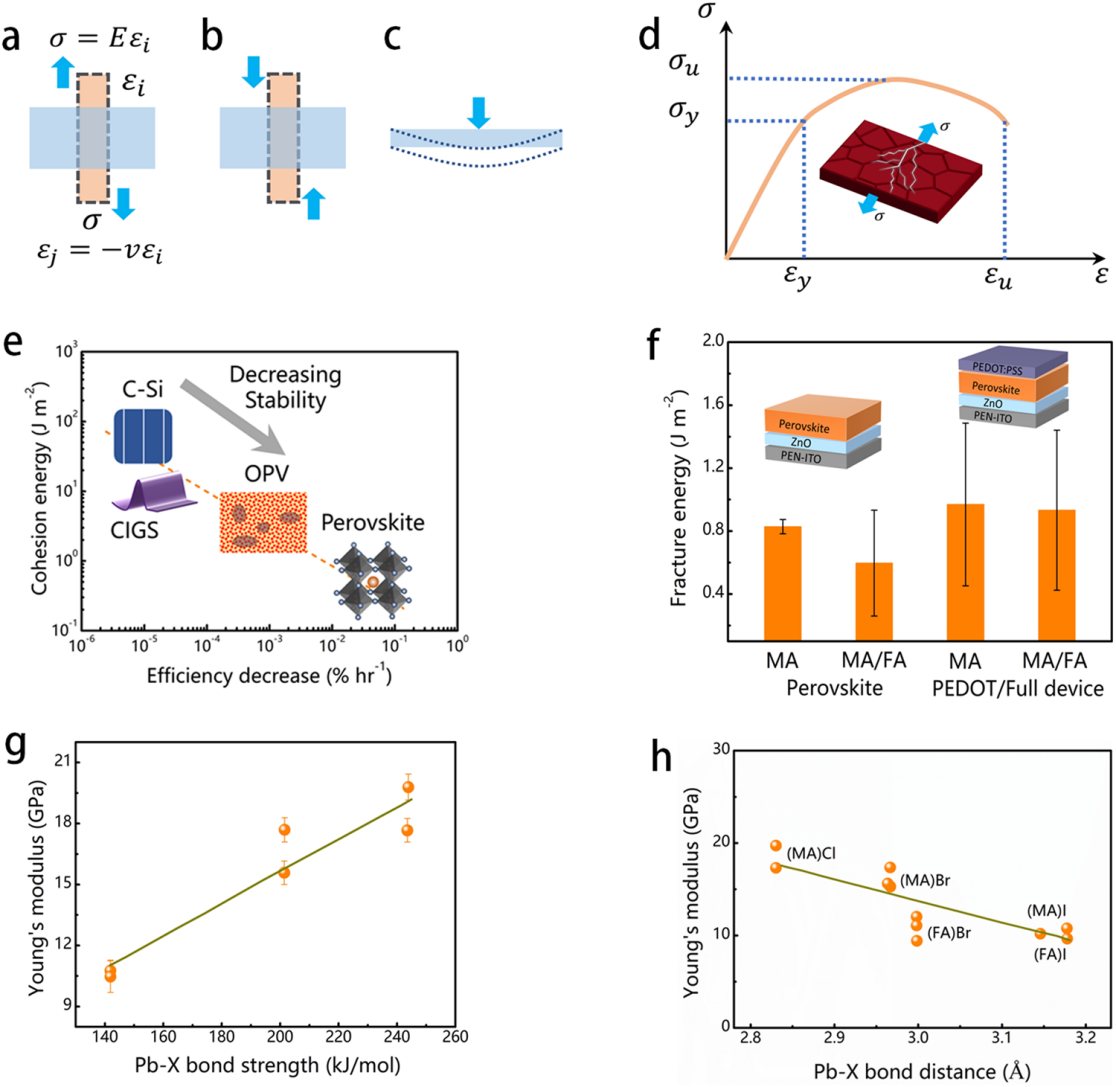
Schematic illustrating some typical loading and failure ways of perovskite layers, including **a** tension, **b** compression, and **c** bending. **d** The stress–strain behavior of materials displays a progression from elastic to plastic deformation, culminating in fracture failure (the inset in (**d**) shows cracks that occur in perovskite under stress). **e** The measured cohesion energy as the function of the degradation rate for different photovoltaic active materials [62]. **f** The fracture energy of perovskites on flexible substrates [62]. Young’s modulus of perovskite films as a function of **g** Pb-X bond strength [63] and **h** the Pb-X bond distance [64]

Elasticity is a characteristic of solid material that allows it to restore its original form once the external stress is removed. This external stress can take various forms, such as tensile (Fig. 3a), compressive (Fig. 3b), and bending stress (Fig. 3c). Elastic deformation takes place prior to the plastic yield or mechanical breakdown of materials and is commonly observed in strain-induced stability problems with perovskite materials frequently experiencing distortion or crack (Fig. 3d). Therefore, in order to understand the mechanics-coupled stability for perovskite materials, it is necessary to first address the structure–performance relationship regarding their elastic properties. Young’s modulus (*E*) is a critical parameter for evaluating the flexural endurance of flexible materials. Therefore, an experimental study should be conducted to measure the long-term operational stability of organic–inorganic halide metal perovskites using Young's modulus. Perovskite films form the residual stress during process procedures, causing the formation of cracks and delamination (the inset of Fig. 3d).

The cohesion energy (*G*_*c*_) is a critical parameter for assessing the flexural endurance of devices and is correlated with reliability through deformation and manufacturing processes in various photovoltaic devices. The development of interfacial residual stresses due to fabrication processes can result in the amplification of these stresses during operation, leading to the evolution of defects within photoelectronic materials. The determination of *G*_c_ is based on the strain energy release rate, while the estimation of *G*_c_ is linked to the critical stress (*P*_c_) causing crack development. Equation 1 is utilized for the computation of *G*_c_ [65].

1 $${G}_{c}=\frac{12{{P}_{c}}^{2}{a}^{2}}{{B}^{2}{E}^{,}{h}^{3}}{\left(1+0.64\frac{h}{a}\right)}^{2}$$

By utilizing the compliance relationship in Eq. 2, the elastic compliance measurement of d*Δ*/d*P* is employed to experimentally ascertain an estimation of the crack length as shown following:

2 $$a={\left(\frac{\mathrm{d}\Delta }{\mathrm{d}P}*\frac{B{E}^{,}{h}^{3}}{8}\right)}^{1/3}-0.64*h$$

The mechanical stability of perovskite materials is characterized via Eq. 3 [66]:

3 $${k}_{IC}=0.016{\left(\frac{H}{E}\right)}^{-1/2}\frac{P}{{c}^{3/2}}$$

where *P* is the applied stress, *H* is the hardness, *E* is Young's modulus, and *c* is the length of the crack.

As shown in Fig. 3e, the fracture energy for perovskite devices [62, 67] is less by an order of magnitude than of OPV and c-Si or CIGS solar cells by two orders of magnitude [68]. Nonetheless, residual stress plays a crucial role in determining the stability of perovskite photovoltaics. By using the slot-die coating technique, the *G*_c_ of methylammonium (MA) perovskite was determined to be 0.83 ± 0.04 J m^−2^. Similarly, the perovskite layers obtained by spin-coating technology at various ratio of MA_0.17_FA_0.83_ showed a comparable result with a *G*_c_ of 0.56 ± 0.10 J m^−2^ [62]. The decrease in *G*_c_ in MA/FA-based components, when contrasted with the MA-based perovskite, may be attributed to the relationship between fracture strength and perovskite grain size. Smaller-grained films have larger grain boundaries, which behave as defects and weaken the quality of layers, making them susceptible to crack propagation under mechanical loads (Fig. 3f).

The chemical bonding of Pb-X is considered a crucial parameter in determining the flexural endurance of perovskite devices [69]. The Pb-X bond strength increases in the order of *E*_I_ < *E*_Br_ < *E*_Cl_, which consistent with the increasing Young’s modulus (Fig. 3g) [63]. Moreover, the size, symmetry, and electronegativity variations among the organic cations may have an impact on Young’s modulus of the halide perovskites. For instance, the M-X bond length in the inorganic framework is typically longer in FA^+^-based halide perovskites than in their MA^+^ counterparts because of larger FA^+^ than MA^+^ [64, 70]. Therefore, the high-stiffness perovskite films can be produced by A-site cations. The conventional photoactive perovskite material, CH_3_NH_3_PbI_3_ (MAPbI_3_), has Young’s modulus in the range of 14 ~ 35 GPa (calculated values) dependent on the various phases. Figure 3h indicates longer Pb-X bond distance which leads to lower mechanical stiffness across the family of lead-based single halide perovskites. The Young’s modulus (*E*, *G*, and *B*) shows a high dependence with the metal-halide bond strength, exhibiting a tendency of from I, Br, to Cl for perovskite materials with the same A cation and metallic ions [64].

## Merits and Shortcomings of F-PSCs

**Fig. 4:**
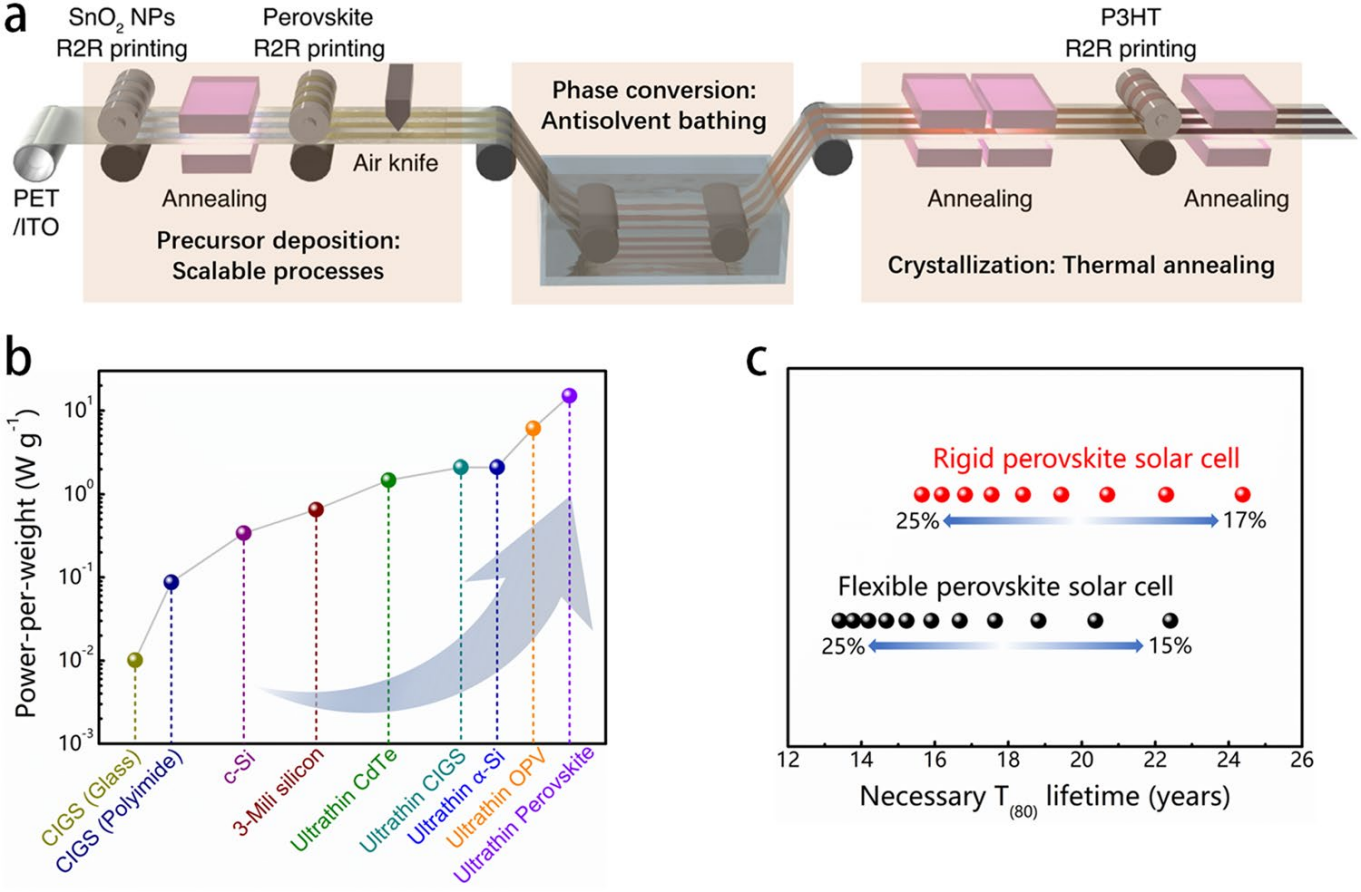
**a** Schematic diagram representing Roll-to-Roll processing of F-PSCs [25]. **b** Comparison of the published power-per-weight performance of lightweight solar cells [72]. **c** Direct comparison of rigid/flexible PSCs with the commercial c-Si devices in terms of their necessary lifetimes for a given efficiency to achieve the same cost as c-Si [73]

F-PSCs possess distinctive merits such as arbitrary-shaped forming, roll-to-roll manufacture, high power-per-weight, and flexibility compared to rigid photovoltaics. Roll-to-roll deposition technology is a convenient and efficient manufacturing process for mass-producing large-area F-PSCs. It offers benefits such as cost-effectiveness, efficient material usage, and high production capacity. The rate of roll-to-roll deposition in solution phase chemistries for F-PSCs depends on the evaporation of solvents and film transformations, which are commonly carried out in ovens [26]. Developing routes to reduce the required time for these processes can further enhance the cost efficiency of F-PSCs. The roll-to-roll process enables fast and affordable production of flexible and lightweight PSCs, making them suitable for various wearable electronics, portable power supplies, and BIPV [38, 71]. Thus, the advancement of the roll-to-roll coating process or printing process has enabled the transfer of flexible photovoltaics from the laboratory to the industrial sector for large-area device manufacturing. To achieve roll-to-roll production of F-PSCs, it is significant to realize low roughness and large-scale production of sequential functional layers. Although the roll-to-roll fabrication of charge transport layers has already been established in OPVs, it is not fully explored in the process of perovskite layers yet. Three essential steps involved in the roll-to-roll deposition of perovskite layers are listed as follows: firstly, the deposition of perovskite precursor solution onto flexible substrates with scalable coating techniques. Secondly, the removal of solvents by heating or vacuuming, for example, converts the wet films from the precursor state into a state of super-saturation. Lastly, thermal annealing is employed to facilitate the crystallization procedure (Fig. 4a) [25]. The transition from a wet precursor state to an intermediate phase is crucial in the vast production of high-grade perovskite films. For instance, the technique of anti-solvent extraction has been widely employed to achieve excellent performance in small-sized photovoltaic cells (PSCs) by inducing a state of super-saturation through the rapid drip of anti-solvent while spin-coating process. The redundant solvent is eliminated via spin-coating, which creates a supersaturated film from the precursor wet film containing a retardation mediator.

Furthermore, lightweight and outstanding flexibility is critical for power sources in electronics like portable power supply. The mechanical durability of perovskite materials is strongly dependent on substrates. Therefore, the plastic substrates have a fundamental impact on the lightness and flexibility for perovskite devices. For example, the highest recorded power-per-weight of 23 W g^−1^ was achieved by the lightweight F-PSCs [72]. Moreover, serval transparent electrodes made of plastics substrates have been explored to substitute the natural fragile ITOs for F-PSCs (Fig. 4b). The feature of high power-per-weight for F-PSCs is very advantageous when compared to other power-generating devices including motors (0.3–8.4 W g^−1^) and heat pumps (0.03–10 W g^−1^). Furthermore, the low-temperature solution-processable methods could further reduce the cost of F-PSCs modules. It was suggested that utilizing lightweight plastic substrates can effectively reduce the total cost of PSCs installation as compared to rigid ones, leading to a lower comprehensive cost that permits different mounting methods [73]. Additionally, the latest research also commented that a rigid PSC with a 17% PCE would require a minimum of 24 years to become competitive with silicon installed in a residential area. However, F-PSCs with the same efficiency would need to endure for 19 years only [73]. Therefore, according to the anticipated balance of system cost one could expect by 2030, a perovskite photovoltaic module with an efficiency of over 23% would last for 24 years if the substrates are rigid, but reduced to 17 years for flexible substrates (Fig. 4c). Nonetheless, so far, the F-PSCs have not exhibited excellent flexural endurance and environmental stability compared with those rigid one. The primary cause for the poor property of devices could be specifically attributed to the difference between rigid glass and flexible plastic substrates in terms of the coefficient of thermal expansion and transmittance of materials. The limited thermal-annealing process for flexible substrates impedes the production of metallic oxides and charge transport layers, thus lowering the PCE and operational stability. Moreover, plastic substrates usually exhibit lower optical transmittance compared to rigid glass substrates, which is another critical factor for the limited photovoltaic property of F-PSCs.

## Strategies Utilized in Flexible Perovskite Solar Cells

### Internal Stress Engineering

**Fig. 5:**
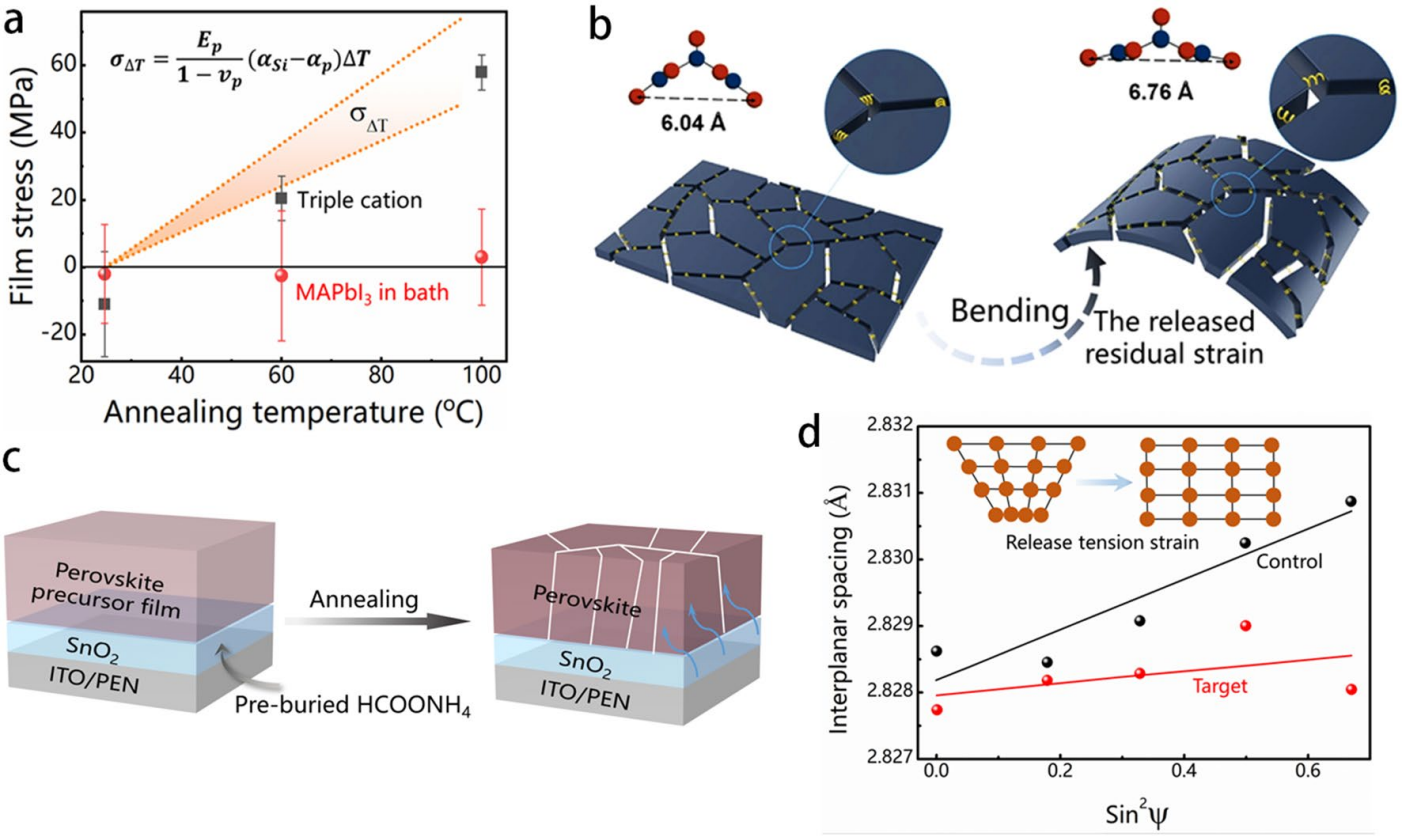
**a** The stress levels of CsMAFA-triple cation (black) were measured at different temperatures and the comparison to the predicted stress levels. The stress levels of MAPbI_3_ (red) were measured after formation at room temperature and with annealing at different temperatures, showing low-stress values in all cases [77]. **b** Schematic illustration of a grain boundary stress release strategy for F-PSCs [78]. **c** Schematic diagram of releasing the residual strain and micro-strain of perovskite films through the pre-buried HCOONH_4_ additive [79]. **d** The plots of lattice spacing *d*_(012)_ versus sin.^2^Ψ for perovskite films with the pre-buried HCOONH_4_ additive [79]

Thermoplastic polymer-based materials, such as PEN, PET, and PI, are mostly used as plastic substrates of F-PSCs due to their outstanding flexibility, roll-to-roll processability, and lightweight properties [74–76]. However, these flexible substrates become deformed and soften during a repeated bending deformation and/or thermal-annealing process, resulting in an inhomogeneous stress distribution among the perovskite layer. This has a tremendous influence on the mechanical stability and flexural endurance of F-PSCs. Figure 5a presents the residual stress against annealing temperature for perovskites, in which the anti-solvent exchange agent of diethyl ether was used in material fabrication. The films exhibit tensile stress of 57.6 ± 4.9 MPa annealing at 100 °C, while those present a value of 20.7 ± 6.6 MPa at 60 °C. There is a residual stress of -10.8 ± 15.2 MPa for perovskite films fabricated without annealing (*i.e*., all processes were performed at 25 °C). A clear linear relationship was observed between the residual tensile stresses in perovskite layers and the annealing temperature, showing the stress values increased at higher annealing temperatures [77]. The correlation can be determined by computing the anticipated stress caused by the difference in thermal expansion, ${\sigma }_{\Delta T}$, given by Eq. 4:

4 $${\sigma }_{\Delta T}=\frac{{E}_{p}}{1-{v}_{p}}{(\alpha }_{S}-{\alpha }_{p})\Delta T$$

where* E*_p_ represents the modulus of the perovskite, *ν*_p_ denotes the Poisson's ratio of the perovskite, while *α*_s_ and *α*_p_ correspond to the thermal expansions of the substrate and perovskite, respectively. To note here, that negative stress was observed in films, indicating the presence of residual solvent and incompletely converted perovskite compounds. This was supported by the fact that the photocurrent density was slightly lower for F-PSCs (25 °C) compared to those annealed at higher temperatures. The stress-annealing temperature linearity demonstrated that no stress release occurs within the temperature range. Therefore, the stress distribution in the perovskite layer has a remarkable effect on the device's stability.

The molecule internal stress control strategy has been widely used to diminish residual stress and enhance the flexural endurance of F-PSCs. Wang et al. [78] proposed a stress release engineering technology to achieve high mechanical stability and phase stability of F-PSCs via introducing a cross-linkable additive with 3D architecture in perovskite grain boundaries (Fig. 5b). The optimized devices obtained a record efficiency of 21.63%, which could retain 91.8% of the initial efficiency after 10,000 bending cycles at a radium of 5 mm. In addition, F-PSCs have been severely restricted due to the interfacial residual stress resulting from the significant deformation of flexible substrates. Liu et al. [79] utilized an amine-based organic molecule additive as a pre-buried molecule in tin dioxide (SnO_2_) to achieve excellent mechanical stability in F-PSCs, retaining over 90% of its initial PCE values after 4000 bending cycles at a bending radius of 7 mm. This can be attributed to the additionally enhanced interfacial adhesion in perovskite films (Fig. 5c). Moreover, the (012) crystallographic plane of the perovskite film, without HCOONH_4_ in SnO_2_ ETL, exhibited a shift toward a lower 2θ position as the Ψ angle was varied from 0° to 55° using GIXRD technology. The lattice spacing *d*_(012)_ increases monotonically, indicating significant tensile stress within the perovskite film. Conversely, the lattice spacing *d*_(012)_ keeps constantly at different depths for the perovskite film treated with HCOONH_4_. This result suggests the residual stress had been released effectively. This study indicates that the HCOONH_4_ can significantly improve the perovskite lattice homogeneity in the top layer (Fig. 5d). The developed F-PSCs exhibited a record efficiency of 22.37% and maintained it over 90% after 4,000 bending cycles. A nanocellular scaffold was designed that serves as an interfacial layer to build a flexural buffer layer, resulting in released mechanical stresses during the distortion of the flexible devices [80]. These results demonstrate that the nanocellular scaffold buffer layer enhances the mechanical stability of F-PSCs due to the uniform distribution of internal stress in the perovskite layer.

### Grain Boundary Modification

**Fig. 6:**
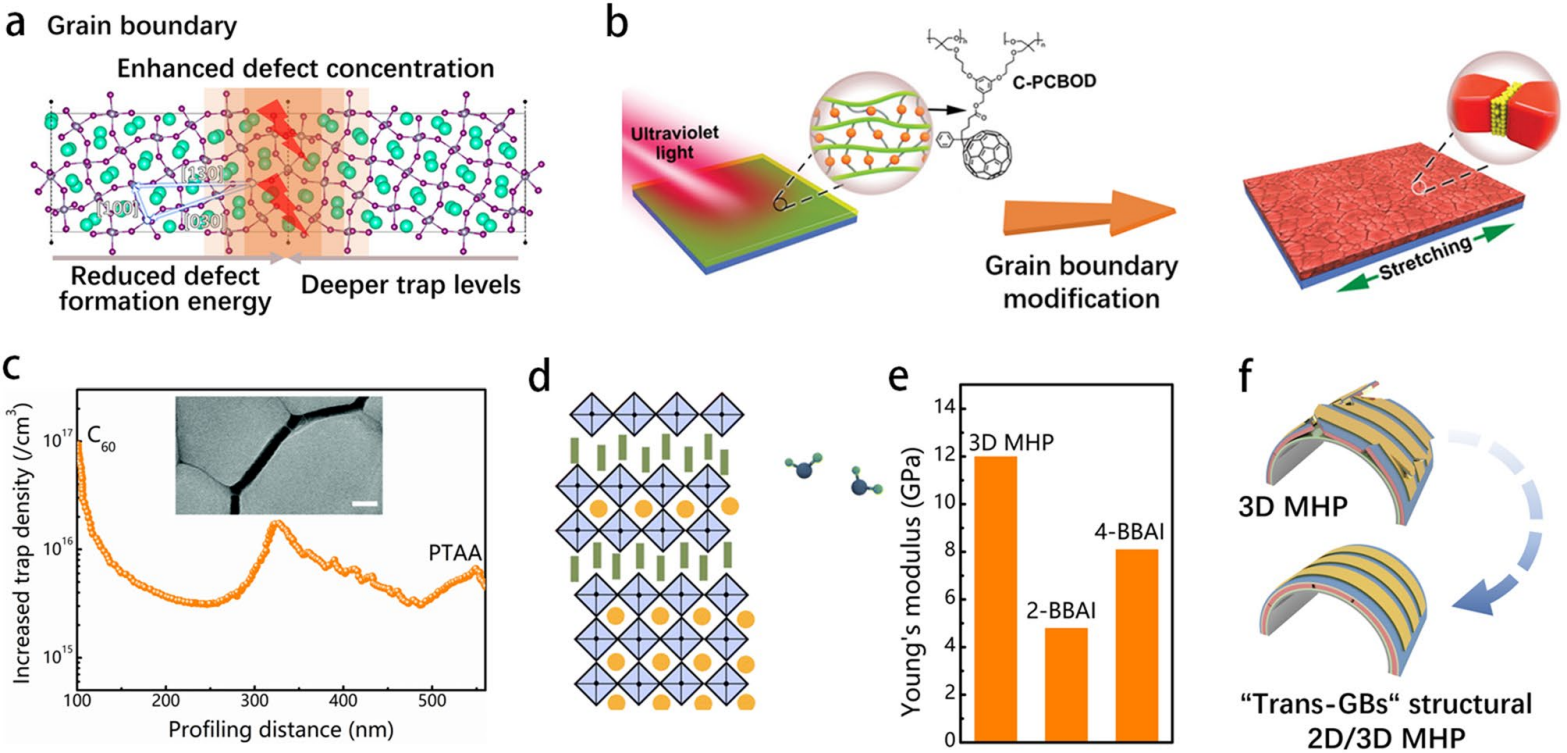
**a** Atomic model of the symmetric tilt grain boundary. The boundary of CsPbI_3_ cells is depicted by the solid lines, whereas the grain boundaries are represented by the dashed lines [84]. **b** The in situ polymerization of cross-linked [6, 6]-phenylC_61_-butyric oxetane dendron ester (C-PCBOD) for covering the region among grain boundaries [83]. **c** Spatial distribution of the defect density of perovskite film after bending cycles [55]. **d** Illustration of the grain boundary modification of 4-bromobenzylammoniumiodide 2D perovskite on the 3D perovskite surface based on moisture-guided growth [85]. **e** Average Young’s modulus of 3D MHP, 2-BBAI, and 4-BBAI films [85]. **f** Schematic illustration of the flexural endurance enhancement effect of 2D perovskite on the F-PSCs [85]

Typically, the low crystallinity of perovskites is typically attributed to a large number of grain boundaries (GBs), which generate deep-level trap states in the bandgap of semiconductors, resulting in an enhanced in carrier recombination rate and a significant reduction in open-circuit voltage. Similarly, flexible perovskite films contain internal defects, particularly at the grain boundary, which decreases the mechanical stability of F-PSCs (Fig. 6a) [54, 81, 82]. Notably, in a status of bending, stretching, or twisting, GBs are the stress concentration region for the perovskite films. The deformation damage is hard to recover via conventional routes, thus leading to the weak comprehensive performance of F-PSCs. Currently, grain boundary modification is an effective strategy to prevent phase transitions and eliminate the detrimental trap states, improving the flexural endurance stability of F-PSCs. To address this issue, many strategies on grain boundary modification and dimensional engineering have been utilized to enhance the flexural endurance of F-PSCs. For instance, the utilization of cross-linkable organic molecules has been applied to decrease trap-state density and thus improve perovskite crystallinity and film quality, resulting in an enhancement of the mechanical performance of flexible photovoltaics. A photo-polymerized C_61_-based organic molecule [83] was utilized as a grain boundary modification agent to passivate the defects via the cross-linked polymerization of C_60_-based organic molecules initiated with ultraviolet light (254 nm) and obtained the extraordinary mechanical stability of perovskite films (Fig. 6b).

To investigate the spatial distribution of defect states in devices, the width of depletion layers was varied in devices with different DC voltages in capacitance–voltage (*C–V*) characterization. Subsequently, the capacitance value was also measured at each voltage to determine the corresponding charge density (*N*_c_) for each edge of depletion layers with the following Eq. 5 [86]:

5 $$N_{c} \left( X \right) = - \frac{2}{{e\varepsilon \varepsilon _{0} A^{2} }}\left[ {\frac{{d\left( {1/C^{2} } \right)}}{{dV}}} \right]^{{ - 1}}$$

By subtracting *N*_c_ before and after bending, the spatial distribution of the increased defect density of devices can be acquired. As illustrated in Fig. 6c, the device exhibited a higher increase in defect density close to the C_60_ layer, which could be attributed to large strain in the area. Furthermore, the higher proportion of enhanced defect density near 350 nm may be linked to the higher concentration of GBs in the horizontal direction (Fig. 6c) [55]. In addition, theoretically dimensional engineering could improve the flexural endurance of 3D perovskites. Shi et al. [54] reported a dimension engineering strategy for accurate growth of two-dimensional perovskites at the grain boundaries of three-dimensional perovskites, while a trans-GBs structure was formed under the stimulation of water molecule, thus resulting in a mechanically stable F-PSCs (Fig. 6d). Based on the results obtained from the PeakForce QNM imaging technique, the 2-bromobenzylammonium iodide (2-BBAI) capping layer can be considered as softer as evidenced by its lower Young’s modulus (4.8 GPa) in comparison with 3D MHP (12 GPa), which is summarized in Fig. 6e. Such type of capping layer can be difficult to reinforce the flexural endurance of perovskite films. Conversely, the 4-BBAI-based trans-grain boundary two-dimensional phase has an average Young’s modulus (8.1 GPa) similar to that of 3D MHPs (12 GPa). Therefore, it can effectively reinforce the GBs, ultimately improving the fracture energy of the entire perovskite film (Fig. 6f). The polymer-based additives used for grain boundary modification, such as polyvinylpyrrolidone (PVP) [87], polyvinyl alcohol (PVA) [88], and polyurethane (PU) [89], can improve the flexural endurance of perovskites and even stitch cracks at the GB regions, thus improving the flexible devices’ stability performance. A soft repairing route [90] was proposed for GBs, and a stretchable sticky elastomer (s-ELA) was used to join the rigid crystallite grains, thereby resulting in the passivation of the defects at GBs and improving the mechanical endurance of F-PSCs. Moreover, the devices maintained 86% of the initial PCE after 10,000 cycles at 10% stretching, indicating their outstanding stretching durability. Typically, outstanding flexural endurance and high photovoltaic performance cannot be obtained simultaneously based on the polymer-based grain boundary modification strategy. Similarly, small molecular functional additives present unique advantages in passivating grain boundary defects and enhancing the flexural endurance of perovskite film. For example, methylammonium succinate (MS) acts as a multifunctional organic salt, which could release strain and reinforce grain boundaries. This effect could be correlated with the ethylene group betwixt the two carboxyl groups of the MS molecular which provides enough toughness to alleviate the strain [91]. These strategies generally follow the idea of incorporation of organic molecules into GBs and passivating the defects at GBs. However, the interaction of these organic passivation molecules could be too weak to fundamentally guarantee the mechanical stability of F-PSCs. Song et al. [81] introduced a sulfonated graphene oxide (s-GO) to build stable GBs via interacting with the [PbI_6_]^4–^ at GBs. The defects of vacant iodine could be effectively passivated by the s-GO-[PbI_6_]^4–^ complex, achieving excellent mechanical stability. Nonetheless, the perovskite films containing s-GO demonstrate a lower average elastic modulus in comparison with the control samples.

### Self-healing Strategy

**Fig. 7:**
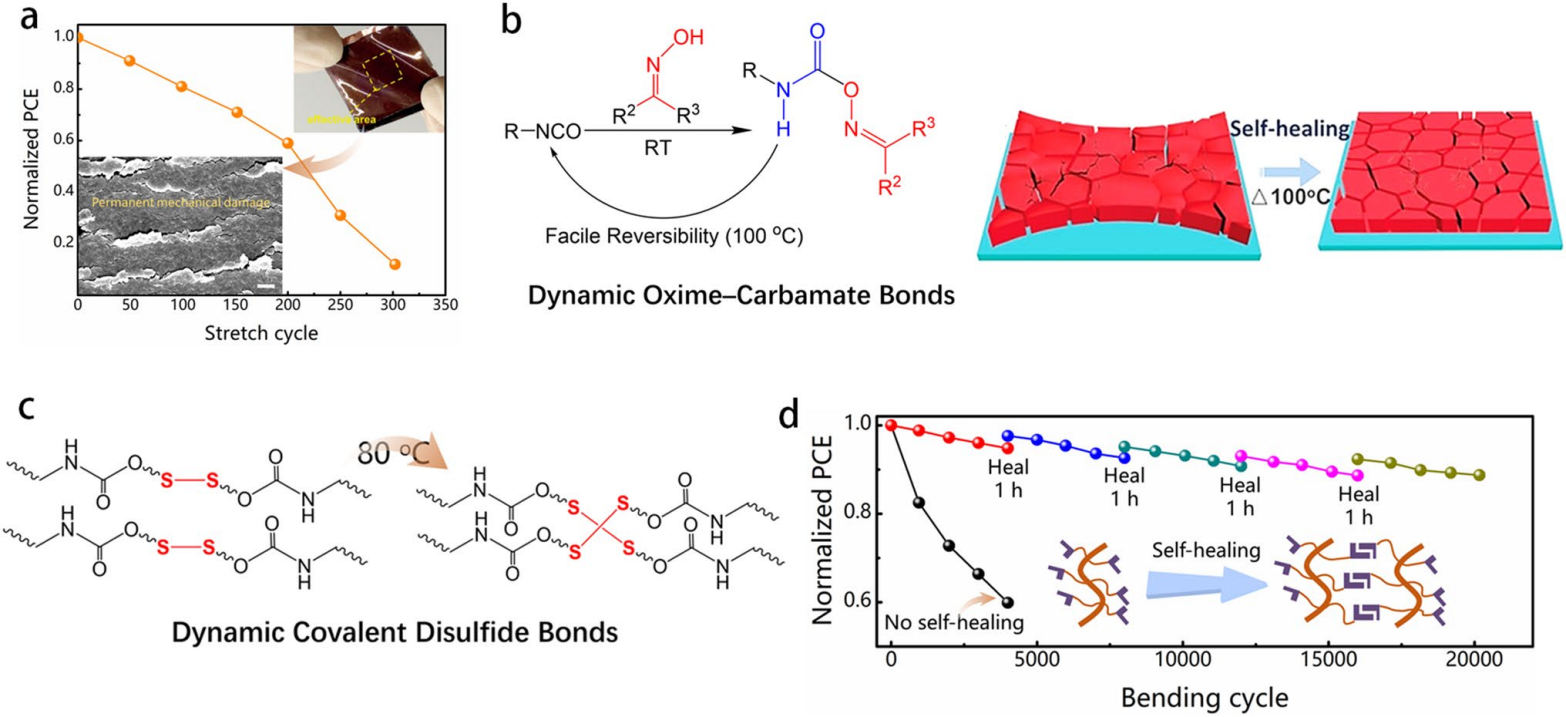
**a** Normalized PCE of F-PSCs without self-healed function as a function of the number of stretching cycles. Inset: illumination of cracked flexible perovskite film and F-PSCs after being stretched [96]. **b** Synthesis and thermal-driven self-healing mechanism of the self-healing PU [96]. **c** Schematic diagram of the thermal-driven self-healing process of perovskite films with dynamic covalent disulfide polyurethane elastomers [92]. **d** Stability test of the F-PSCs with/without self-healing function [97]

Achieving high-performance F-PSCs with long-term operational stability requires considerable attention to deployment on novel materials including perovskite films, electrodes, and flexible substrates. The main objective is to create effective and stable flexible photovoltaics that can withstand repeated mechanical displacement. Despite massive efforts made in this line, current F-PSCs still suffer from the crack formation during deformation (Fig. 7a). Subsequently, irreversible performance degradation occurs along with mechanical fracture [92–95].

Recently, many protocols for implementing the self-healing strategy for F-PSCs have been suggested to solve the problem of rapid attenuation and irreversible recovery of photovoltaic performance caused by repeated bending deformation processes. For example, a self-healing polymer has been proposed to mix into the perovskite precursor as a polymer scaffold for perovskite crystallization and to stitch cracks on the perovskite surface. The self-healing mechanism of perovskite films includes either physical or chemical behaviors, such as *van der* Waals contacts [98], dynamic covalent behavior [99], the supramolecular effect [100], and chemical bonding [101]. Integrating the self-healing materials into GBs of perovskite films equipped them with self-healing abilities. The adverse variables regarding stability can be transformed into advantages due to repairing damaged F-PSCs. A self-healing polyurethane (s-PU) scaffold was introduced with dynamic oxime-carbamate linkages into perovskite films to obtain self-healed F-PSCs via a thermal annealing treatment at 100 °C to enhance the mechanical durability of perovskites [96]. Due to the repaired cracks and passivation of the GBs of the perovskite film via the self-healing s-PU molecule, the F-PSCs retained over 88% of their initial PCE after 1000 stretching cycles (Fig. 7b). Nevertheless, the F-PSCs typically operate at room temperature, which implies that the thermal self-healing process may potentially harm the organic carrier transport layers. Therefore, it is vital to create a self-healing perovskite that can heal itself under gentle external stimuli. The disulfide groups in polyurethane are weak covalent bonds, which can assist to offer a healing functionality at lower temperature and hold a proper level of bond strength as well. The restructured disulfide bonds can easily form free radicals at low temperature (~ 60 °C). Thus, the sulfur radicals create disulfide bonds with the neighbors via an exchange reaction as cooling down the temperature to implement the self-healing function (Fig. 7c). 88% of the initial PCE of the damaged F-PSCs was recovered with this method [92]. A cross-linkable additive [97] was prepared to achieve ultra-high bending durability and efficient self-healing F-PSCs at room temperature. The cross-linked monomer located on the perovskite GBs could potentially release residual stress in 3D perovskite films. In addition, it can heal deformation-induced cracks in the perovskite due to the covalent disulfide bonds. The F-PSCs containing cross-linked monomer exhibit excellent self-healing ability (Fig. 7d). Inspired by the scales of the pangolin, a biomimetic self-healing route via introducing a soft elastomer of diphenylmethane diisocyanate polyurethane (MDI-PU) in perovskite films to achieve a flexural endurance flexible device was demonstrated. The resultant F-PSCs retained 87.8% of its initial PCE values after 2000 bending cycles [89]. Mechanical damage caused by repeated deformation could be healed multiple times under specific conditions, greatly extending the lifespan of flexible devices and reducing their costs.

Moisture is typically considered a negative factor that affects the stability of perovskite. A mechanically stable formamidinium lead iodide (FAPbI_3_) film using a moisture-triggered self-healing process was fabricated [88]. This process allows for the repair of mechanical damage in a humid environment. The poly(vinyl alcohol) micro-scaffold can absorb water molecules and sew up cracks in brittle perovskite films. During this self-healing process, the loosely bonded halide ions interact with a thermal source or ultraviolet inducer, leading to the ion migration and creation of interstitial and vacancy defects. Therefore, the pursuit of a mild self-healing technology is of great importance in enhancing the mechanical property of F-PSCs. By strengthening the understanding of the materials and interfaces employed, it is confident to produce F-PSCs that are flexible and suitable for commercial use.

### Crystallization Regulation

**Fig. 8:**
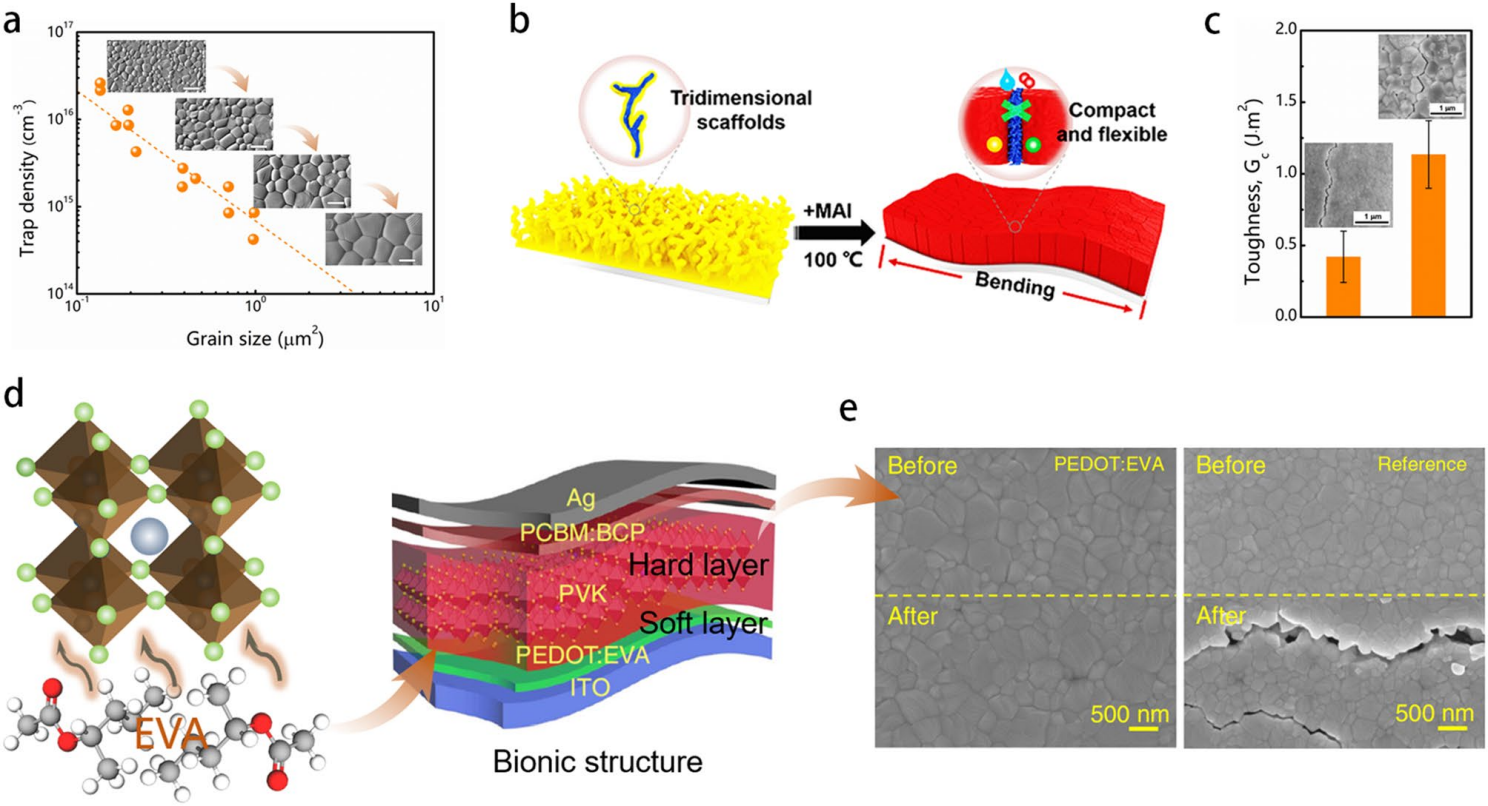
**a** The dependence of density of defects in perovskite films with grain size [102]. **b** The enhancement in solvent evaporation by low-boiling-point additive methyl methacrylate molecules to promote the formation of mesoporous self-polymerized methyl methacrylate in PbI_2_ film [103]. **C** The toughness of small-size grained MAPbI_3_ thin film and MAPbI_3_/ETL interface in large-size grained MAPbI_3_ thin film [104]. **d** Biomimetic crystallization mechanisms of the flexible perovskite film [53]. **e** Microstructures for the perovskite films before and after bending [53]

The flexural endurance of F-PSCs is closely correlated with the crystalline quality of perovskite on flexible substrates. In case of poor crystalline quality, the formation of numerous grain boundaries occurs, resulting in lower mechanical stability and reduced flexural endurance for the perovskite film. As the grain boundaries are prone to point defect formation, which may serve as deep trap levels. The trap density is expected to increase as grains become smaller because of a greater overall grain boundary area. The simulation results suggest that the effective density of traps increases significantly, surpassing the anticipated rise from the grain boundary area alone [102]. It can be inferred that perovskite films with large grains are far more stable than those of small grains (Fig. 8a). The flexural endurance and mechanical stability of F-PSCs were granted by an in situ self-polymerization of methyl methacrylate (sMMA) in PbI_2_ to form a distinctive autonomously longitudinal organic scaffold (Fig. 8b). The perovskite crystals with vertical crystal growth can be confined within an sMMA scaffold that fills the pinholes and cracks within perovskite films and thus enhance the mechanical stability [103]. The toughness of the interface between MAPbI_3_ and ETL is demonstrated by its corresponding fracture energy, which is approximately three times higher at 1.14 ± 0.24 J m^−2^. This indicates that the fracture occurs along the MAPbI_3_/ETL interface. The results demonstrate that larger grains are advantageous in enhancing the total fracture resistance of perovskite films for PSCs, leading to improved mechanical stability and reliability (Fig. 8c).

Inspired by the alternating soft-hard structure of the spine, an adhesive polymer between the ITO and perovskite layers was employed for the same purpose [53]. This approach facilitates the oriented crystallization of the perovskite and improves the adhesion between the perovskite layer and the flexible substrates as well (Fig. 8d). When bending the samples, negligible cracks were found on the perovskite films based on PEDOT:EVA layer, while obvious cracks were determined on the films upon PEDOT:PSS layer. The Young’s modulus of PET/ITO/PEDOT:EVA HTL film surface (139 MPa) was determined to be lower than that of PET/ITO/PEDOT:PSS (258 MPa). In short, the excellent perovskite crystallization process effectively enhances the films’ mechanical stability (Fig. 8e). The F-PSCs showed exceptional mechanical stability, maintaining over 85% of their original PCE even after 7,000 narrow bending cycles without significant angular dependence. However, this method only provides limited control over the top portion of the perovskite layer and poses challenges in terms of repeatability and production scalability. Moreover, the pinholes that originate from the crystallization process of perovskite film are potentially responsible for creating a slightly increased stress concentration area within F-PSCs, which consequently leads to a decrease in flexural endurance and mechanical stability [44, 105–107]. To accomplish the outstanding long-time operational stability of F-PSCs, it is essential to reduce pinholes and increase the crystallization quality of perovskite films by controlling the nucleation and crystallization rate. To date, a variety of techniques have been employed to manufacture high-quality perovskite films, including the use of additives to delay the crystallization rate and anti-solvent to make pinhole-free dense perovskite films. Though additives are beneficial in improving the crystalline quality, an excessive amount of additives to perovskite precursors often brings on precipitation due to interaction among them, creating numerous pinholes and poor-quality crystalline films. Therefore, it highly requires to develop a novel and universal method that can fabricate high-quality, pinhole-free perovskite films to achieve exceptional mechanical robustness of F-PSCs. The cross-linking reaction is usually triggered after the perovskite film has already formed, resulting in an inability to precisely monitor and guide the evolution of the perovskite film in real time. A meticulously constructed functional monomer as an in situ cross-linked molecule shows the simultaneous activation of the in situ cross-linking and perovskite growth process to produce perovskite films of large grains, dense stacking, and a preferred crystal orientation [48].

## Flexible Electrode Materials for F-PSCs

The primary objective in the advancement of F-PSCs is to investigate transparent conductive electrodes that demonstrate high conductivity. The ultimate goal for the bottom electrode is to exhibit exceptional characteristics, including transparency, flexibility, conductivity, low-temperature processability, and chemical stability. The bottom electrode, referred to as the window electrode, plays a crucial role in attaining high performance in bottom-illuminated F-PSCs by enabling the transmission of incident light for absorption by the perovskite layer, while simultaneously facilitating the collection of photogenerated charges. Transparent conductive oxides (TCOs), conductive polymers, carbon nanomaterials, and metallic nanostructures are widely employed as transparent electrodes in F-PSCs. Transparent metal oxide electrodes on plastic substrates, predominantly ITO, are commonly used in F-PSCs due to their excellent photoelectronic performances. Unfavorably, on a plastic substrate, the ITO tends to crack because of the substrate shrinkage during mechanical bending, leading to considerable sheet resistance and diffuse reflectance [108–111]. Thus, it is vital to develop flexible transparent electrodes with properties of outstanding mechanical stability and high transparency and conductivity. Several materials, including carbon-based nanomaterials, metal mesh, transparent conducting polymers, and metal nanowires are employed in F-PSCs. In this instance, it is critical to balance the conductivity and transparency of the flexible electrodes. Moreover, for an ideal F-PSC, it should retain a minimum of 90% of its initial PCE even after undergoing 1,000 bending cycles to meet the commercialized requirements [112]. The ‘cask effect’ of F-PSCs remains a barrier to their deployment in practical applications. Furthermore, the stability of F-PSCs depends strongly on the physical performances of their flexible substrates, including the glass transition temperature, thermal expansion coefficient, and water and oxygen barrier performance, as shown in Table 2 [113].

**Table 2:** Performance parameters for conventional flexible substrates

Substrate	PET	PEN	PI	PDMS	PC
Modulus (MPa)	2 ~ 4 × 10^3^	0.1 ~ 0.5 × 10^3^	2.5 × 10^3^	1	2.0 ~ 2.6 × 10^3^
T_g_ (°C)	70 ~ 110	120 ~ 155	155 ~ 270	125	145
T_m_ (°C)	115 ~ 258	269	250 ~ 452		115 ~ 160
Water absorption (%)	0.4 ~ 0.6	0.3 ~ 0.4	1.3 ~ 3	> 0.1	0.16 ~ 0.35
Work Temp. (°C)	− 50 ~ 150		< 400	-45 ~ 200	-40 ~ 130
Vol. Res. (Ω.cm)	10^19^	10^5^	1.5 × 10^17^	1.2 × 10^14^	10^16^ ~ 10^18^
Thermal expansion coefficient (ppm/°C)	15 ~ 33	20	8 ~ 20	310	75
Density (g/cm^3^)	1.39	1.36	1.35 ~ 1.43	1.03	1.20 ~ 1.22

**Fig. 9:**
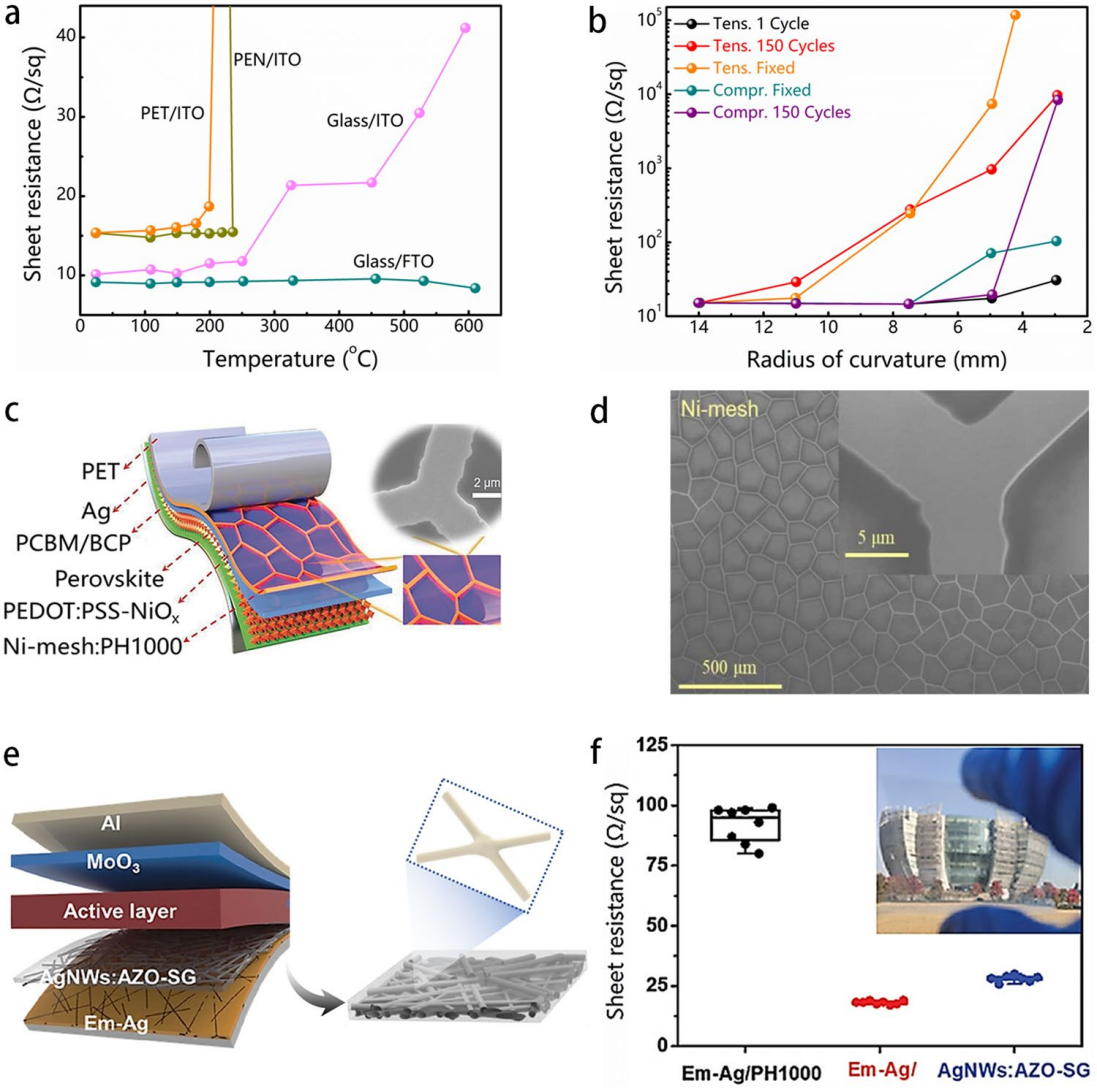
**a** The sheet resistance of flexible/rigid ITO electrodes varies with temperature changes [114]. **b** The sheet resistance of PEN/ITO electrodes varies with the radius of curvature changes under stress [114]. **c** Schematic illustration of a flexible photovoltaic device with Ni-mesh: PH1000 hybrid electrode. **d** High-magnification SEM images of Ni-mesh [120]. **e** Schematic illustration of flexible organic solar cells with a hybrid electrode of Em-Ag/AgNWs:AZO-SG [121]. **f** Conductivity statistics of hybrid electrodes. The inset is a picture of Em-Ag/AgNWs:AZO-SG flexible transparent electrode [121]

A summary of the diverse flexible transparent electrodes employed in F-PSCs, along with their optical transparency and electrical conductivity, is presented in Table 3. ITO/PET and ITO/PEN exhibit marginally lower transmittance in the visible-light spectrum than their glass counterparts. However, the transmittance is significantly lower in the ultraviolet range considering the robust absorption of polymer substrates. The ITO/PEN exhibits remarkable thermal stability, as its resistance remains unchanged (15 Ω sq^−1^) even after annealing up to 235 °C. However, an annealing process at over 250 °C results in a significant 20-fold resistance increase, primarily due to the deformation of PEN substrates. The critical temperature for ITO/PET is only 150 °C. A continuous bending of ITO/PET and ITO/PEN films at a curvature radius lower than a certain critical value can cause their degradation (Fig. 9a) [114]. Moreover, the ITO-based flexible substrate would crack when being continuous bent or stretched due to its rigidity and brittleness, leading to a dramatic decrease in its conductivity. Due to the brittleness, the conductivity of ITO degrades as the curvature increases. Under tensile stress, the cracking/channeling becomes the primary cause of failure, while under compressive stress, the debonding may be a factor to be seriously considered. For example, when the r value is less than 14 mm, the ITO-based flexible substrates could generate degradation under tensile stress, while the degradation occurs for r values less than 8 mm under compressive stress (*e.g*., *R*_sheet_ increased by 50% after 150 cycles at r = 5 mm). Typically, the degradation emerges at a critical value for the radius of curvature at around r = 5 mm per cycle (Fig. 9b).

**Table 3:** Performance parameters of conventional flexible transparent electrodes

Flexible electrode	Sheet resistance (Ω sq^−1^)	Transmittance (%)	Refs
PET/ITO	10 ~ 15	73.1	[115]
PEN/ITO	14	78	[116]
PI-SWNT/MoO_*x*_	82	80	[117]
PET/PEDOT:PSS	234.3	> 80	[118]
PET/Ag-mesh/PH1000	3	82 ~ 86	[119]
PEN/graphene/MoO_3_	552.0	97	[42]

Recently, architectural designs that facilitate mechanical deformation have shown the beneficial in reducing damage caused by flexing, indicating significant potential for real-world application of F-PSCs. Especially, metal meshes provide an intriguing substitute for the TCOs electrode used commonly in flexible photovoltaics, owing to their excellent mechanical stability and conductivity. Figure 9c presents a transparent conductive electrode with an embedded metal mesh on a flexible substrate [120]. The conductive network was facilitated by the Ni-mesh with excellent conductivity (Fig. 9d). The PET/Ni-mesh/PH1000 electrode surpasses the Ag-mesh alternative in terms of high transparency (85–87%) while maintaining morphological consistency even in extensively sized PET/Ni-mesh: PH1000 electrodes. After 5,000 bending cycles at a radius of curvature of 5 mm, the fabricated F-PSCs retained 95.4% of the initial PCE.

Silver nanowires (AgNWs) are considered as conductive materials for flexible photovoltaic devices due to high conductivity and inherent flexibility [122–124]. Nevertheless, the AgNWs network obtained through solution processing methods generally exhibits a limited coverage (< 40%), high surface roughness, elevated junction resistance, and poor chemical stability. Consideration should be given to both the substrate and electrode roughness as they affect the morphology of the upper layers. In general, an elevated roughness of the electrode surface can result in a decrease in the crystal quality of the perovskite absorber, thereby weak device performance. Moreover, the junctions of AgNWs concentrate heat locally by radiation which further decreases the device's stability. So far, many post-treatments have been proposed for those AgNWs-based flexible substrates; however, none of them have completely solved the issues faced with flexible devices. A welding technique for an integrated flexible transparent electrode design [121] was reported, showing a low sheet resistance (*R*_sh_) of 18 Ω sq^−1^ and a high transmittance of 95% at 550 nm (Fig. 9e). The resultant welding AgNWs-based flexible electrode has excellent mechanical stability in the bending and peeling tests (Fig. 9f). Furthermore, the corrosion and oxidation of substrates must be considered when AgNWs networks are exposed to air or severe conditions. To provide a passivated surface, the blocking layer must have mechanical durability, superior barrier property, and high transmittance to reduce excess energy loss. Im et al. [125] proposed to protect the AgNWs electrode by vacuum-depositing a 10-nm-thick ITO layer, and the resultant flexible devices exhibited over 14% efficiency and exceptional chemical stability.

## Environmental Stability and Advanced Encapsulation Technologies in F-PSCs

The perovskite absorber is easily damaged by continuous light irradiation, oxygen, and humidity, resulting in crystal structural transformation and degradation of the perovskite absorber layer and further decreasing F-PSCs photovoltaic performance. Long-term light exposure could lead to degradation and lattice distortion because the light-induced degradation process can damage the perovskite structure via a thermal-induced lattice distortion [126, 127]. The ultralow thermal conductivity of perovskite materials (∼ 0.4 W m^–1^ K^–1^) and the conventional organic hole transport materials (∼ 0.15 W m^–1^ K ^–1^) make these two kinds of functional layers fail to effectively dissipate heat by themselves [128, 129]. Subsequently, heat accumulates inside PSCs to accelerate device degradation. To overcome this, Zhou et al. [130] added silica aerogel to a perovskite film to serve as both a heat dissipation medium and a passivator for the perovskite surface. The characterization of integrating infrared thermal imaging and a laser thermal conductivity meter revealed that the additive with higher thermal conductivity improves the stability and thermal transport efficiency of F-PSCs [131]. Furthermore, continuous illumination could promote ion migrations in F-PSCs and thus lead to local chemical component segregation, which would alter mechanical performance distribution. Advanced encapsulation technology is immediately required for F-PSCs with a plastic substrate, as the conventional rate of encapsulation fails to ensure the stability of the devices [132–134].

It is widely recognized that F-PSCs are prone to deterioration when exposed to an atmospheric environment, which poses a significant challenge in creating F-PSCs that can maintain long-term stability in practical applications. Glass has been widely used to encapsulate rigid perovskite solar cells to prevent the spreading of moisture or oxygen into functional layers. However, the rigid and fragile characteristics of glass substrates render them unsuitable for F-PSCs applications. The primary focus of this section is to explore the most recent advanced encapsulation technologies that are being employed to block water and oxygen into the vulnerable perovskite layer, which significantly enhances their long-term operational stability and lifespan.

**Fig. 10:**
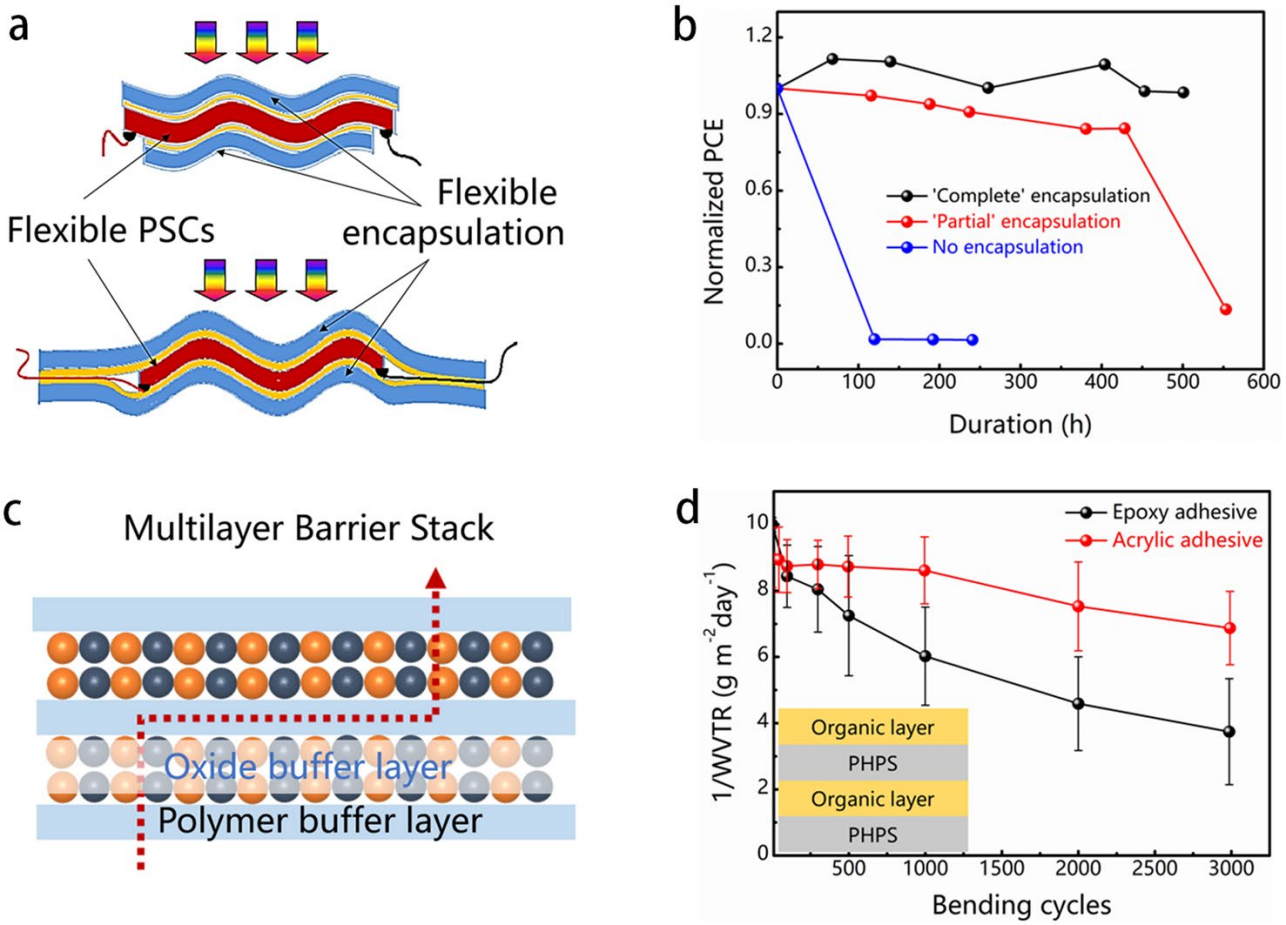
**a** Illustration of a conventional encapsulation structure [137]. **b** Photovoltaic performance dependence of non-encapsulated and encapsulated F-PSCs as a function of storage time under ambient conditions [137]. **c** Schematic diagram of hybrid organic/inorganic multilayer barrier structure. **d** Significant improvement in water-oxygen resistance after incorporating organic interlayers, as shown in the inset featuring the organic/inorganic multibarrier [135]

The evaluation of encapsulation materials usually involves examining their oxygen transmission rate (OTR) and water vapor transmission rate (WVTR) [135]. These measures reflect how fast oxygen gas and water vapor can cross a specified area of the material within a particular period of time. Generally, higher OTR and WVTR levels indicate greater quantities of oxygen gas and/or water vapor entering the sensitive material, leading to a faster degradation [136]. To date, F-PSCs have been encapsulated using either a single-layer or multiple-layer encapsulation technique. The former approach uses only a single layer of inorganic or organic material to encapsulate the F-PSCs. However, pinholes and flaws should probably to form on the surface of encapsulants, allowing oxygen or moisture to infiltrate the encapsulation layer. In the latter approach uses organic/inorganic hybrid thin film by combination inorganic layers with organic layers. Using barrier films and encapsulation to shield the sensitive photoactive layer from moisture and oxygen has proven to be one of the most important and successful methods for stabilizing F-PSCs. Typically, the inorganic encapsulation layer, e.g., Al_2_O_3_, SiO_2_, Si_3_N_4_, or SiO_x_N_y_, acts as a buffer layer to prevent the penetration of water and oxygen. In addition, the organic encapsulation layer, e.g., polyurethane (PU), poly(ethylene-1-octene) (POE), and poly(1,3,5-trimethyl-1,3,5-triviny-lcyclotrisiloxane) (PV3D3), works as a buffer layer, reducing the defects in inorganic encapsulation layer and further improving the reliability of the device (Table 4). Multilayer thin-film encapsulation has been widely employed in organic photoelectric devices. As shown (Fig. 10a), in comparison with the non-encapsulated control devices, the device lifetime has been significantly increased for ‘partial’ and ‘complete’ encapsulated F-PSCs stored under an atmospheric environment. ‘Partially’ encapsulated devices maintained approximately 80% of their initial PCE for over 400 h before a sharp performance decline (Fig. 10b) [137]. The point was revealed when the stacked blocking films under conditions of extensive bending were evaluated [135]. Their research examined the performance of cured perhydropolysilazane (PHPS) as an inorganic encapsulation layer. The WVTR significantly increased throughout an initial cyclic bending test with bending 150 times at a radius of 3 mm due to the natural brittleness of cured PHPS films. Amending this, polymer-based barrier interlayers were introduced to construct a PHPS/polymer multilayer barrier encapsulation on flexible substrates (Fig. 10c) and were able to attain significantly superior mechanical reliability with only a slight decrease in WVTR after 3,000 bending cycles as shown in Fig. 10d. A similar phenomenon [138] was discovered when they subjected eight variations of multi-barriers to a severe cyclic bending test. The integration of polymer interlayers in the barrier architecture significantly improved mechanical robustness.

**Table 4:** The performance and stability of encapsulated devices conducted under different conditions

Encapsulation materials	Perovskite materials	Stability test conditions	% of initial PCE	Refs
AZO Al_2_O_3_	MAPbI_3_	500 h/85 °C	86.7	[139]
PDMS	MAPbI_3_	3000 h/ambient	100	[140]
PV3D3/Al_2_O_3_	(FAPbI_3_)_0.87_(MAPbBr_3_)_0.13_	300 h/50 °C	97	[141]
Organosilicate	MAPbI_3_	3000 h/85 °C	92	[142]
UV-curable fluoropolymer	(FAPbI_3_)_*x*_(MAPbBr_3_)_1−*x*_	2190 h/outdoor test	95	[143]
PI tape	MAPbI_3_	1620 s/water	96.3	[144]
Adamantane nanocomposite	MAPbI_3_	60 s/water	95	[145]
PU	(5-AVA)_*x*_MA_1−*x*_PbI_3_	2136 h/outdoor test	97.5	[146]
UV-curable epoxy	MAPbI_3_	144 h/85 °C	85	[147]
SnO_*x*_	MAPbI_3_	7300 h/60 °C/N_2_	95	[148]
Polyolefin	Cs_0.17_FA_0.83_Pb(Br_0.17_I_0.83_)_3_	1000 h/85 °C	99	[149]
Poly(ethylene glycol)/resin	MAPbI_3_	450 days	100	[150]

## Conclusion and Outlook

F-PSCs have experienced significant advancements in PCE and mechanical stability during the current decade (2013–2023). We summarize the recent breakthroughs achieved in the stability of F-PSCs and attributed them to the compositional engineering of perovskite and flexible transparent conductive electrodes as well as advanced encapsulation technologies. These advancements have resulted in a record PCE of over 23% for flexible perovskite solar cells. Several innovative and effective approaches to date indicate that this field is rapidly developing, which pushes the photovoltaic performance of F-PSCs closely to that of rigid counterparts.

So far, flexural endurance and long-term operational stability remain vulnerabilities in their practical application. The intrinsic brittleness of the perovskite lattice makes it susceptible to distortion, leading to unfavorable defects and cracks in the perovskite films during repeated deformations. To bring these F-PSCs into a large-scale application, it is paramount to induce self-healing capability and push their long-term operational stability, thereby enabling the devices with recoverable lifetimes. Strategies have been applied to enhance the flexural endurance and mechanical stability of F-PSCs, such as component optimization, grain boundary modification, self-healing technologies, crystallization regulation, and interfacial modification. The self-healing behavior of perovskite is induced by external stimulation, which enables flexible devices to achieve recoverable performance and stability.

Enhancing mechanical and environmental stability is a key aspect in the development and large-scale manufacturing of F-PSCs. In order to limit the effect of oxygen and moisture, various barrier materials and advanced encapsulation techniques have been employed, including polymer barrier materials, thin films, and nanoparticle polymer matrixes. The state-of-the-art encapsulation strategies exhibit robust stability in laboratory conditions. Further study is desirable to uncover the effects of oxygen and moisture. At present, a series of advanced encapsulation technologies have been proposed to ensure the environmental stability of F-PSCs, although they are still in the initial stages. Considering other factors that include the surface roughness, composition, and crystallinity of the perovskite film, as well as additive engineering and interface modification, are crucial to improve the long-term operational stability of F-PSCs. Incorporating these considerations alongside encapsulation strategies is recommended to enhance the mechanical and environmental stability of F-PSCs in the future.

There is also a lack of a standard characterization of long-term operational stability. So far, the reports carried out under various conditions are incomparable in parallel and thus fairly provide few useful references for subsequent research. For the industrialization of flexible perovskite photovoltaic devices, it is necessary to establish a scientific evaluation standard procedure on their stability.
